# Progress in the application of motor imagery therapy in upper limb motor function rehabilitation of stroke patients with hemiplegia

**DOI:** 10.3389/fneur.2025.1454499

**Published:** 2025-02-05

**Authors:** Shuying Shen, Tianchen Chu, Jing Wang, Hangyu Zhao, Jinli Tang, Linya Xu, Wei Ni, Liping Tan, Yu Chen

**Affiliations:** ^1^Department of Neurosurgery, Suzhou Ninth People’s Hospital, Suzhou, China; ^2^Suzhou Medical College of Soochow University, Suzhou, China; ^3^Department of Neurosurgery, The Second Affiliated Hospital of Soochow University, Suzhou, China; ^4^Department of Neurosurgery, The Affiliated Hospital of Nantong University, Nantong, China; ^5^Department of Neurosurgery, Yancheng NO.1 People’s Hospital, Yancheng, China; ^6^Department of Neurosurgery, The Second People’s Hospital of Huai'an, Huai'an, China; ^7^Department of Emergency, Suzhou Ninth People’s Hospital, Suzhou, China

**Keywords:** motor imagery therapy, stroke, hemiplegia, motor function, rehabilitation

## Abstract

Stroke is the leading cause of disability in Chinese adults. Upper limb motor dysfunction is a common manifestation of neurological dysfunction after stroke and can exert significant effects on a patient’s daily living ability and quality-of-life. Therefore, it is crucial to provide appropriate rehabilitation treatment for upper limb motor function in stroke patients with hemiplegia. Currently, rehabilitation treatment for upper limb motor function in hemiplegic stroke patients in China includes motor therapy, neuro-promoting technology, occupational therapy, physical factor intervention, speech therapy, and swallowing therapy. Motor imagery therapy has also been shown to effectively promote the rehabilitation of upper limb function in stroke patients. Here, we review the concept, classification, mechanism of action, application, and effect of motor imagery therapy for the rehabilitation of upper limb motor function in stroke patients with hemiplegia in China. We summarize the available evidence, arising from Chinese experience, to support the implementation of this method in medical and rehabilitation institutions.

## 1 Introduction

Stroke is a medical condition in which the blood vessels in the brain suddenly burst or become blocked, thus obstructing blood flow and causing damage to brain tissue (1). There are approximately 13.7 million new stroke patients worldwide each year. In China, the incidence of stroke ranges from 1.2 to 180 per 100,000, resulting in approximately 2 million new cases annually. Stroke is the second leading cause of death, following ischemic heart disease. Stroke is the primary cause of adult impairment in China due to its high incidence, disability, mortality, recurrence, and economic burden (2). Upper limb motor impairment is one of the most common neurological conditions following stroke, exerting significant impact on a patient’s quality of life. Even six months after a stroke, 30 to 60% of patients still exhibit this impairment, which significantly affects their ability to perform daily activities.

Although traditional rehabilitation methods, such as acupuncture, massage, and cupping, have been used to treat upper limb problems, their efficacy remains limited. Over recent years, kinesthetic therapy has emerged as one of the most prominent therapeutic modalities in this field. Motor imagery (MI) therapy involves repeatedly simulating and rehearsing a specific action without producing any obvious motor output. This practice activates the corresponding area of the brain responsible for motor memory, ultimately improving corresponding motor function. MI therapy has been shown to have a positive impact on the ability of stroke patients to regain the use of their upper limbs. The concept of MI originates from mental imagery. In 1950, Hossack introduced the idea of a mental image, which suggests that the central nervous system can produce a response similar to that caused by receptor activation even when the senses are not stimulated. According to Decety et al. (3), MI is a dynamic condition in which a specific motor behavior is internally practiced in memory without any explicit motor output. Using various methods of imagination, MI can be classified into internal MI and external MI. Internal MI is also referred to as kinesthetic imagery (KI). During kinesthetic imagery, an individual focuses on their own sense of movement and visualizes completing the entire action. Furthermore, KI involves using one’s own proprioception as the point of attention and visualizing the completion of the entire movement. External MI, also known as visual imagery (VI), involves visualization of the movement from an external perspective. During VI, the imager assumes the role of a bystander and observes themselves or others in action from a distance. This technique, also known as third-party imagination, relies heavily on visual sensory input and is closely associated with the surrounding environment. Embedded motor imagery (EMI) and added motor imagery (AMI) are two categories of MI that can be distinguished by whether they are used in conjunction with other therapeutic techniques. EMI refers to the application of MI therapy throughout the entirety of the rehabilitation training process and aims to complete different tasks at the same time to increase the imagination component. AMI is separated from other training tasks, and patients complete MI treatment individually and completely by listening to recordings or a therapist’s direct instructions (4). Currently, supplementary kinesthetic imagination is used extensively; this is because this technique is easier to employ than other methods.

In this study, we investigated the use of MI therapy for the rehabilitation of upper limb motor function in stroke patients with hemiplegia. Our research provides reference guidelines for potential future use in domestic medical and rehabilitation facilities.

## 2 Methodology

Using the PubMed, China National Knowledge Infrastructure and Wanfang Data, we performed a narrative evaluation of the existing literature covering peer-reviewed publications published between January 1, 2017, and June 30, 2023. In our initial search, two independent investigators (SYS and TCC) used the key term “motor imagery” in combination with the term “stroke.” The PubMed, China National Knowledge Infrastructure and Wanfang Data databases yielded 1,046 titles and abstracts in total. We limited our selection based on the following inclusion criteria: (1) studies focusing on effect of MI therapy on stroke patients; and (2) articles briefly describe the concept and mechanism of MI. Two authors (SYS and TCC) independently examined every record in the electronic database based on the previously stated eligibility criteria. Articles containing simply abstracts and redundant research were removed. Disagreements between the two authors were resolved by discussion or, if required, by JW, a third author who was not engaged in the data collecting procedure.

**Figure 1:**
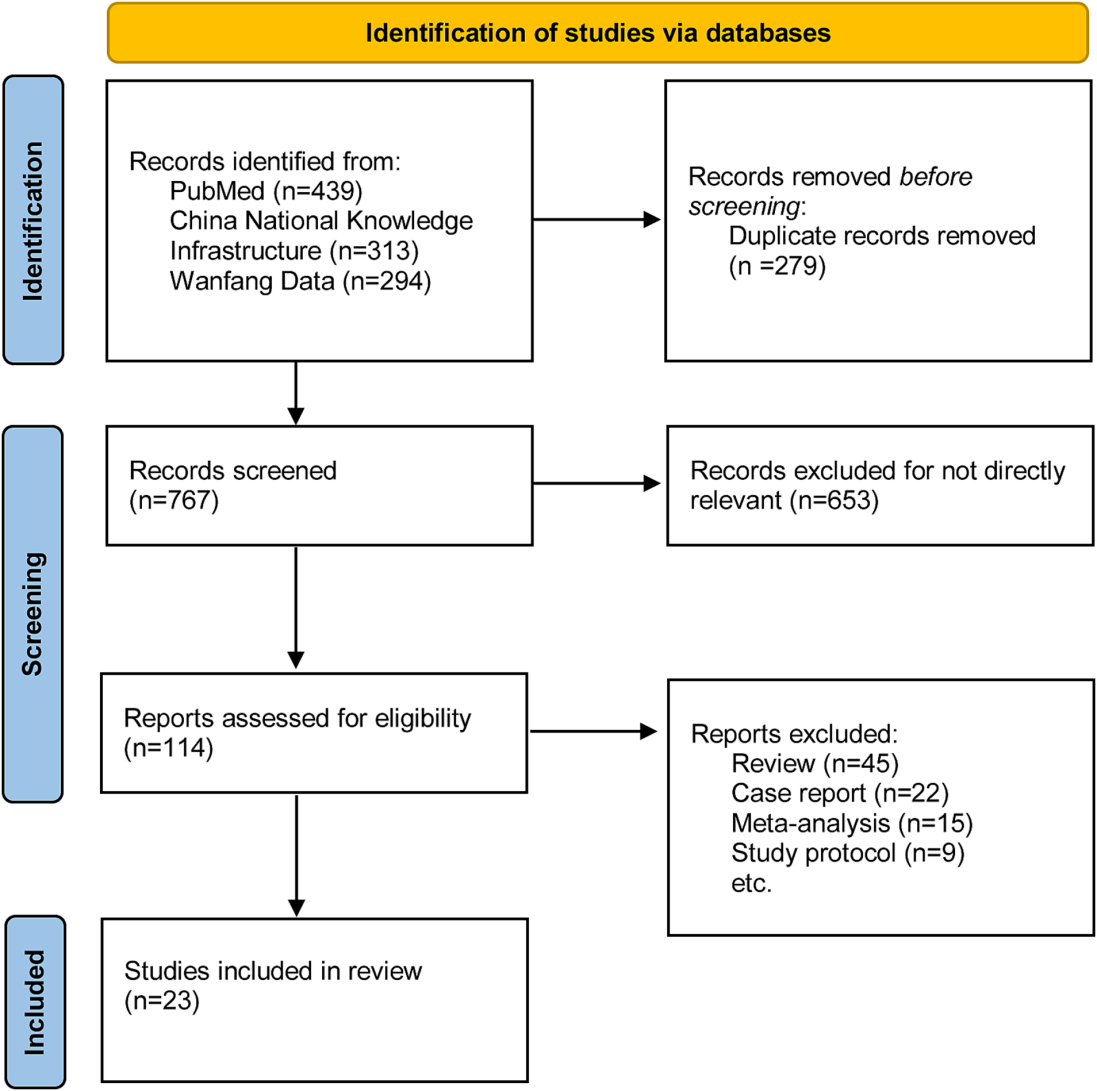
The study search, selection, and inclusion process.

Following a quick examination, 114 entire articles were evaluated for eligibility after 932 pieces were eliminated for being redundant or irrelevant. Due to their failure to fulfill publication type standards, 91 publications were excluded, including 45 reviews, 22 case reports, 15 meta-analyses, and nine study protocols. Finally, 23 studies were included in the present review. The detailed screening process was shown in Figure 1. After meticulous selection and evaluation, all data from the included studies were extracted as follows: basic information including age, gender, study period, evaluation criterion, duration of the disease, specific MI interventions.

## 3 Results

### 3.1 The mechanism of MI and its applicable population

The neural mechanisms of MI are currently unclear. The specific mechanisms underlying the application of MI may include the following two aspects: (1) use-dependent cortical organization and (2) overcoming non-use of the paretic upper extremity. A small number of studies have examined whether cortical organization is a result of MI involvement; in our motor imagery study, functional Magnetic Resonance Imaging (MRI) data post-intervention showed a reduction in the activation of the contralateral primary motor and parietal regions, while increased activation was observed in the ipsilateral primary motor, premotor, and supplementary motor areas. The behavioral techniques used in the mental practice protocol contribute to the efficacy of the protocol in overcoming the impact of limb non-use (5). However, MI has been widely used for patients with Parkinson’s disease to regulate the frontal attention system (6, 7). The prevailing theory is the neuroplasticity-based psychoneuromuscular theory. This theory suggests that the brain creates and stores a ‘flow chart’ when individuals think about or engage in physical activity; this is known as the psychoneuromuscular theory (PM) model. If the flow diagram used in the activity is the same as the one in MI, then MI reinforcement can strengthen the flow diagram. This means that MI training can cause a neurophysiological response similar to the actual movement. According to the PM theory, the motor plan or “flow chart” of exercise has been stored in the person’s central nervous system, and the patient’s thoughts can help to promote a close connection between the cerebral cortex and the central nervous system, which in turn controls movement (8, 9). Although stroke patients have residual limb dysfunction, most patients with mild syndrome still have the ability to perform MI; moreover, the “flow chart” is partially or completely preserved. Previous studies have proved that the brain compensates for motor deficits through functional recombination after stroke, and that functional recovery after local brain injury depends on the plasticity of the cerebral cortex and the regions that are not affected by the functional neural network (10, 11).

Currently, both domestically and internationally, stroke patients with upper limb impairment must meet the following requirements in order to receive MI treatment (12): (1) aged 18–80 years; (2) patient met the criteria for a stroke as defined by the Fourth National Conference on Cerebrovascular Diseases, supported by head CT or MRI; (3) initial cerebrovascular disease, with a 2- to 3-month clinical course; (4) the completion of kinesthesia and visual imagination questionnaires, and a mini mental state examination (MMSE) (13) (yielding a score > 24 points); (5) limb motor impairment in one of the arms; (6) muscle tone of the affected upper limb ≤ level 2 on the Modified Ashworth Spasticity Rating Scale; (7) motor function of the affected upper limb was recovered, and the muscle strength of the proximal limb was ≥1 level on manual muscle strength examination, and (8) the upper section of Fugl-Meyer assessment (FMAUE) on the Fugl Meyer Motor Function Scale was ≤50 points. Patients were excluded for the following reasons: (1) previous history of stroke, brain tumor, brain injury, or other neurological and psychiatric disorders; (2) cognitive impairment (MMSE ≤22 points), sensory aphasia, or spatial neglect of the affected side; (3) clearly felt pain, swelling, and restricted range-of-motion in the upper limbs from various diseases; (4) Signs of heart failure or unstable angina pectoris, uncontrolled high blood pressure, or severe obstructive pulmonary disease.

### 3.2 Application models of MI

**Figure 2:**
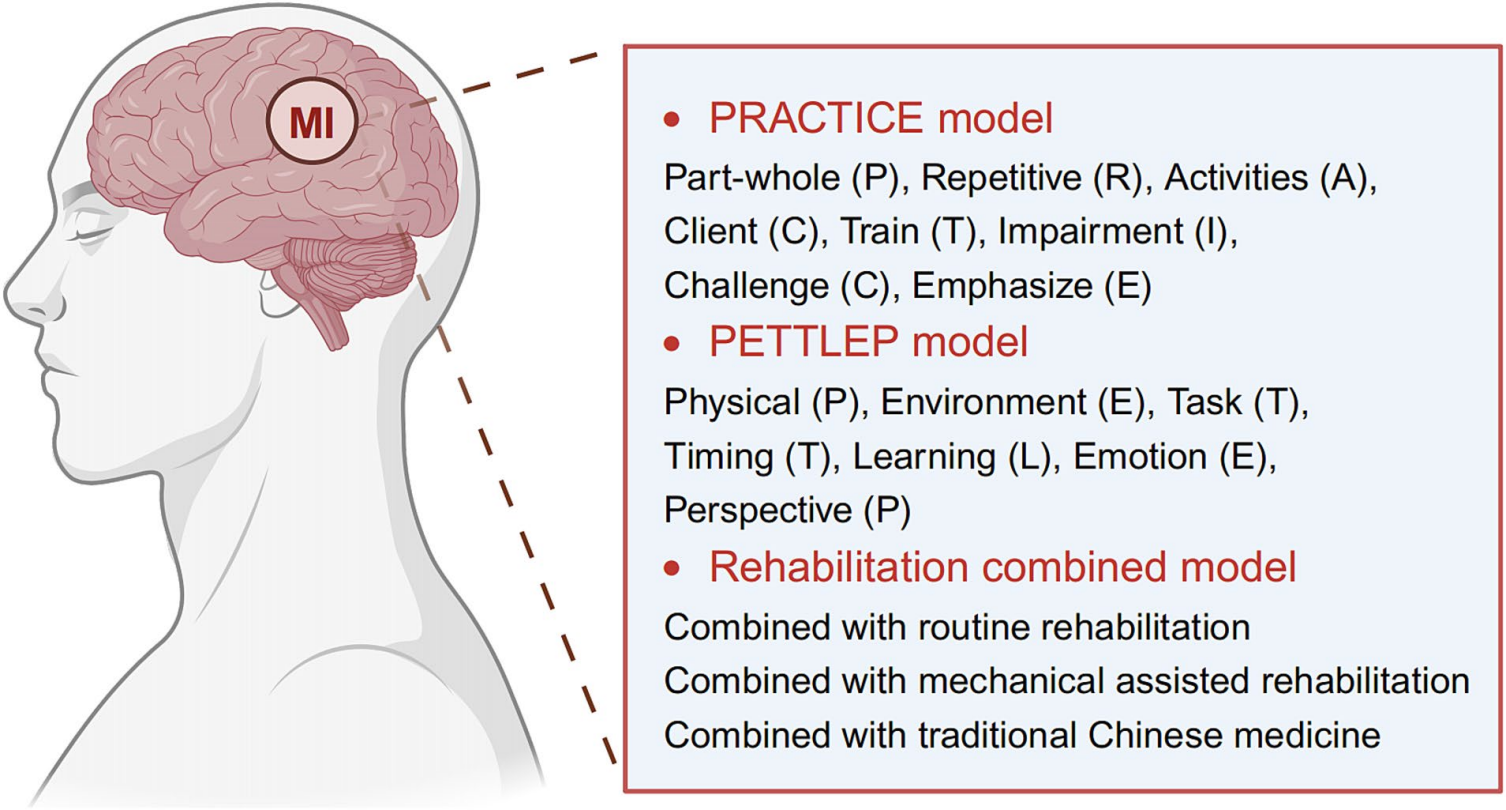
Current main strategies for implementing motor imagery (MI) therapy. Image created with BioRender.com, with permission.

Currently, there are three main strategies for implementing MI (Figure 2): MI based on the PRACTICE model, MI based on the PETTLEP model, and MI mixed with other rehabilitation modalities.

#### 3.2.1 MI based on the PRACTICE model

Page et al. (5) summarized the implementation of an MI scheme as “PRACTICE” (Part-whole, Repetitive, Activities, Client, Train, Impairment, Challenge, Emphasize) and summarized specific training methods for sports imagination. Patients are instructed to sit quietly for approximately 30 min while listening to a motor imagining tape. During the initial three to five minutes of the recording, patients are instructed to visualize themselves in a warm and comfortable atmosphere while relaxing their muscles. For the following 20 min, they are asked to imagine performing the required action. Over the final three to five minutes, patients are encouraged to focus on the present moment, practice calm breathing, and discontinue their imagination. This process lasts approximately 30 min and is repeated once a day, three times a week, for ten weeks.

#### 3.2.2 MI based on the PETTLEP model

Based on the idea of maximizing functional equivalency, Anuar et al. (14) suggested the PETTLEP model (Physical (P), Environment (E), Task (T), Timing (T), Learning (L), Emotion (E), and Perspective (P)). The PETTLEP model for sports imagery more precisely plans a patient’s awakened condition, bodily stance, external stimuli, tasks, and imagination time. To engage the patient’s healthy side, the experimenter performs each new motion before asking them to imagine performing it. This allows the patient to experience the sensation of the limb moving while also exercising their imagination. Finally, the patient is instructed to mentally recreate the movement with their damaged hand after first imitating and comprehending it with their healthy arm. This procedure is conducted once a day, five times a week, for three weeks, and lasts 20 min. The PETTLEP paradigm can render MI therapy more focused, timely, and effective. Analysis showed that the PETTLEP model for MIT significantly improved upper limb motor function in stroke patients when compared to conventional MIT (15).

### 3.3 Application of MI therapy for the rehabilitation of upper limb motor function in stroke patients with hemiplegia

However, there is no set rule for how often to train, how long each training session should be, or how long MI therapy should be applied. Despite this, diverse implementation strategies have been shown to be helpful for the rehabilitation of upper limb function in stroke patients, according to numerous studies. For the best functional recovery, researchers are increasingly combining MI with other forms of rehabilitation treatment procedures. The primary combination treatments are given in Table 1, and described below.

**Table 1:** Motor imagery in conjunction with other rehabilitation techniques.

Study	Centers	Treatment group (No. of participants)	Age range	Male (%)	Mean age ± SD (year)	Study period	Outcome events	Time since stroke (week)	Specific MI interventions	Research summary
Motor imagery combined with routine upper limb function rehabilitation exercise										
Pengpeng et al. (16)	Single	Physiotherapy+ Occupational therapy (15) vs. GMI+ Physiotherapy+ Occupational therapy (15)	40y -75y	Physiotherapy+ Occupational therapy: 80% GMI+ Physiotherapy+ Occupational therapy: 73%	Physiotherapy+ Occupational therapy: 60.7 ± 9.0 GMI+ Physiotherapy+ Occupational therapy: 62.6 ± 10.1	4 weeks	FMA-UE, Brunnstrom, BBT	Physiotherapy+ Occupational therapy: 22.3 ± 7.3 GMI+ Physiotherapy+ Occupational therapy: 22.4 ± 10.8	30 min/time, once/d, 5d/week	Graded motor imagery therapy combined with occupational therapy can effectively improve upper limb motor function in stroke patients.
Siwei et al. (17)	Single	MI+ mCIMT (20) vs. mCIMT (20)	30y -70y	MI+ mCIMT: 70% mCIMT: 55%	MI+ mCIMT: 42.7 ± 8.4 mCIMT: 45 ± 9.1	4 weeks	FMA-UE, ARAT, MBI	MI+ mCIMT: 9.6 ± 3.7 mCIMT: 8.9 ± 3.8	20 min/time, once/d, 5d/week	Motor imagination combined with modified compulsory motor therapy can significantly improve the upper limb and hand motor function and the ability of daily living in stroke patients.
Motor imagery combined with mechanical assisted rehabilitation										
Yanping et al. (25)	Single	Physiotherapy (31) Physiotherapy+ MI (31) Physiotherapy+ MI+ tDCS (32)	≤63y	Physiotherapy: 65% Physiotherapy+ MI: 55% Physiotherapy+ MI+ tDCS: 56%	Physiotherapy: 53.9 ± 8.9 Physiotherapy+ MI: 52.3 ± 10.7 Physiotherapy+ MI+ tDCS: 54.3 ± 8.2	8 weeks	FMA, FTHUE, MBI	Physiotherapy: 34.8 ± 22.9 Physiotherapy+ MI: 36.5 ± 20.6 Physiotherapy+ MI+ tDCS: 36.2 ± 19.9	40 min/time, twice/d, 6d/week	MIT combined with intramuscular adhesive can significantly improve upper limb motor function and daily living ability of stroke patients, and reduce abnormal upper limb tone compared with conventional treatment.
Fangfang et al. (21)	Single	Upper limb exercise (63) vs. Upper limb exercise + MI (63)	18y -85y	Upper limb exercise: 54% Upper limb exercise+ MI: 52.4%	Upper limb exercise: 62.7 ± 5.1 Upper limb exercise + MI: 62.8 ± 5.2	4 weeks	FMA-UE, UEFT, Brunnstrom, iEMG	Upper limb exercise: 42.7 ± 10.2 Upper limb exercise + MI: 42.5 ± 10.1	10 min/time, once/d, 5d/week	The rehabilitation treatment of stroke hemiplegic patients with motion imagination combined with rehabilitation robot can improve the patients’ ability of daily living activities, and also improve the iEMG value of upper limb deltoid, triceps and forearm extensor muscles.
Yuehua et al. (19)	Single	Physiotherapy (37) Physiotherapy+ Upper limb exercise (36) Physiotherapy+ Upper limb exercise +MI (36)	≤85y	Physiotherapy: 62% Physiotherapy+ Upper limb exercise: 72% Physiotherapy+ Upper limb exercise +MI: 69%	Physiotherapy: 68.1 ± 10.6 Physiotherapy+ Upper limb exercise: 65.7 ± 11.3 Physiotherapy+ Upper limb exercise +MI: 69.4 ± 9.6	8 weeks	FMA, MBI	Physiotherapy: 22.4 ± 5.8 Physiotherapy+ Upper limb exercise: 19.5 ± 7.3 Physiotherapy+ Upper limb exercise +MI: 21.3 ± 6.2	15 min/time, twice/d, 7d/week	Rehabilitation exercise with motor imagination combined with upper limb trainer can promote the recovery of upper limb function and improve the ability of daily living in stroke patients.
Yiqian et al. (20)	Single	BCI + MI (12)	20y -75y	BCI + MI: 67%	BCI + MI: 55.1 ± 13.2	4 weeks	FMA-UE, ARAT, BI, MEP	BCI + MI: 11.1 ± 3.7	30 min/time, once/d, 5d/week	Brain-computer interface based on motor imagination combined with multi-modal perceptual feedback training may effectively improve the recovery of upper limb motor function in patients with severe hemiplegia after stroke.
Leilei et al. (24)	Single	Physiotherapy (30) Physiotherapy+ MI (30) Physiotherapy+ MI+ rTMS (30)	18y -70y	Physiotherapy: 53% Physiotherapy+ MI: 63% Physiotherapy+ MI+ rTMS: 43%	Physiotherapy: 49.5 ± 5.5 Physiotherapy+ MI: 44.3 ± 6.6 Physiotherapy+ MI+ rTMS: 46.1 ± 6.8	4 weeks	FMA, FTHUE-HK, CL, CMCT	Physiotherapy: 13.5 ± 2.4 Physiotherapy+ MI: 14.0 ± 3.9 Physiotherapy+ MI+ rTMS: 14.6 ± 3.5	20 min/time, once/d, 5d/week	Both MIT therapy alone and rTMS induced MIT therapy can improve the upper limb motor function of stroke patients, and the effect of rTMS induced MIT therapy is more obvious.
Motor imagery combined with traditional Chinese medicine										
Litai et al. (26)	Single	acupuncture therapy (18) MI (18) acupuncture therapy + MI (18)	39y -70y	Acupuncture therapy: 67% MI: 61% Acupuncture therapy + MI: 56%	Acupuncture therapy: 54.6 ± 12.5 MI: 53.9 ± 13.0 Acupuncture therapy + MI: 53.2 ± 14.2	30 days	FMA, MBI	Acupuncture therapy: 49.0 ± 35.4 MI: 47.9 ± 33.1 Acupuncture therapy + MI: 45.6 ± 33.7	15 min/time, once/d, 6d/week	Three-dimensional acupuncture, motor imagination therapy and their combination can significantly improve the motor function and activities of daily living in stroke patients during recovery, and the combination of three-dimensional acupuncture and MIT is significantly better than the single use.
Wenhai et al. (27)	Single	TEAS (43) MI (40) TEAS+MI (41)	40y -80y	TEAS: 67% MI: 63% TEAS+MI: 63%	TEAS: 63.9 ± 11.0 MI: 61.1 ± 12.3 TEAS+MI: 62.0 ± 10.1	8 weeks	FMA, ARAT, MBI, ADL	TEAS: 50.4 ± 13.1 MI: 51.6 ± 13.69 TEAS+MI: 50.0 ± 12.6	20 min/time, once/d, 5d/week	Longer time of exercise imagination combined with TEAS therapy can better promote the rehabilitation of upper limb motor function in stroke patients.
Yanping et al. (28)	Single	Physiotherapy (31) Physiotherapy+ MI (31) Physiotherapy+ MI+ Kinesio taping (30)	≤56y	Physiotherapy: 58% Physiotherapy+ MI: 55% Physiotherapy+ MI+ Kinesio taping: 60%	Physiotherapy: 50.1 ± 12.6 Physiotherapy+ MI: 52.0 ± 13.9 Physiotherapy+ MI+ Kinesio taping: 52.0 ± 11.5	8 weeks	FMA-UE, FTHUE-HK, MBI, MAS	Physiotherapy: 40.7 ± 25.9 Physiotherapy+ MI: 43.5 ± 20.3 Physiotherapy+ MI+ Kinesio taping: 41.6 ± 22.4	8 min/time, twice/d, 6d/week	tDCS combined with motor imagery therapy can significantly improve the upper limb function of stroke patients with hemiplegia, and it is more obvious than conventional occupational therapy and motor imagery therapy.

GMI, graded motor imagery; FMA-UE, Fugl-Meyer assessment for upper extremity; BBT, box and block test; mCIMT, modified constraint-induced movement therapy; MI, motor imagery; ARAT, action research arm test; MBI, modified Barthel index; FMA, Fugl-Meyer Assessment; BCI, brain-computer interface; BI, Barthel index; MEP, motion evoked potential; UEFT, upper extremity function test; iEMG, integrated electromyogram; rTMS, repetitive transcranial magnetic stimulation; FTHUE, functional test for the hemiplegic upper extremity; FTHUE-HK, Hong Kong version of the functional test for the hemiplegic upper extremity; CL, cortical latency; CMCT, central motor conduction time; TEAS, transcutaneous electrical acupoint stimulation; ADL, activities of daily living; MAS, modified Ashworth scale.

#### 3.3.1 MI combined with routine upper limb function rehabilitation exercise

Gu et al. (16) described the use of a rehabilitation method for 15 stroke patients incorporating MI, mirror movement observation, and mirror movement imitation. These patients underwent daily routines of physical therapy, routine rehabilitative exercises, and graded MI for 30 min. Evaluations of the recovery of upper limb motor function were performed before and four weeks after therapy. For 20 patients who had first-episode strokes, Qu et al. (17) conducted routine rehabilitation training and enhanced mandatory exercise therapy based on MI. Coercive motor therapy (CIMT) can enhance the motor function of the affected limb by restricting a patient’s physical activities and forcibly using the damaged limb (18). This type of rehabilitation training was conducted regularly, twice a day for 30 min each time. Additionally, exercise treatment was required once a day, lasting for 1 h, and motivational interviewing was conducted once a day, lasting for 20 min. Exercises were practiced five days a week for four weeks. Functional evaluations were carried out at enrollment and four weeks after therapy.

#### 3.3.2 MI combined with mechanical assisted rehabilitation

Xu et al. (19) described forty stroke patients who received upper limb trainer rehabilitation exercises along with MI. These patients used an Upper Extremity Exerciser for rehabilitation for 15 min each time after 15 min of MI therapy and 2 min of rest. Patients underwent rehabilitation exercises twice a day with MI combined with the upper limb trainer for a period of eight weeks. Analysis indicated that the combination of the upper limb trainer and motor imagery rehabilitation training promoted the recovery of upper limb function in stroke patients and improved their daily living abilities. In a hospital environment, Hu et al. (20) treated 12 chronic stroke patients for which brain-computer interface (BCI) based on MI and multi-modal perceptual feedback training were added to standard rehabilitation training. BCI technology allowed signals in the training process to be recorded and fed back immediately. All subjects received additional MI-based multimodal perceptual feedback training coupled with a BCI for 30 min per session, five times per week for a total of four weeks. This was performed in addition to their regular rehabilitation therapy. Analysis indicated that a BCI based on motor imagery combined with multi-modal sensory feedback training may effectively improve the recovery of upper limb motor function in post-stroke patients with severe hemiplegia. After their condition had stabilized, Zhang et al. (21) trained 63 stroke patients continuously for four weeks, performing isobaric passive training, assisted motor training, active training, and resistance training once per day and five times per week. After the upper limb robot’s recuperation, MI was performed. MI was performed constantly for four weeks, once per day, five times per week. The rehabilitation treatment of stroke hemiplegic patients with MI combined with a rehabilitation robot can improve daily living activities, and also improve the imaging electromyography (iEMG) value of the upper limb deltoid, triceps and forearm extensor muscle. Repetitive transcranial magnetic stimulation (rTMS), which applies low-frequency rTMS on the MI region in the healthy hemisphere, has been shown to improve the recovery of upper limb function after stroke by reducing abnormal cortical excitability, balancing the neural network, and reducing abnormal cortical excitability (22, 23). In addition to normal rehabilitation, Ju et al. (24) combined rTMS induction (1 Hz MI region of healthy cortex) with MI therapy for 30 patients. The rTMS stimulation frequency was 1 Hz, the intensity was 90% of the resting movement threshold, the continuous stimulation time was 600 s, the interval time was 1 s, and the total number of stimulation pulses was 1,200; each treatment lasted 20 min, and was performed once a day, five times a week, for four consecutive weeks. Each patient received ten treatments in total, or two courses. When comparing these two treatment methods, both MIT therapy alone and rTMS-induced MIT therapy was shown to improve upper limb motor function in stroke patients, with rTMS-induced MIT therapy showing a more pronounced effect. MI was given concurrently with transcranial magnetic therapy, once a day, five days a week, for four consecutive weeks for ten as a treatment course, or a total of two courses, for 20 min each time. Motor-evoked potentials (MEP), cortical latency (CL), and central motor conduction times (CMCTs) were measured before and four weeks after therapy. Transcranial direct current stimulation (tDCS) uses a microcurrent to control the activity of nerve cells in the cerebral cortex to make certain parts of the injured brain more excitable and to aid in the recovery of upper limb function. In a previous study, Zhou et al. (25) treated 32 stroke patients with tDCS in conjunction with MI by utilizing the direct current stimulation mode at a stimulation intensity of 2.0 mA and a stimulation current density of 0.057 mA/cm^2^. The stimulation sites involved an anode in the anterior central gyrus on the other side of the paralysis and the cathode in the opposite shoulder. Treatment was performed for 20 min on each occasion, once a day, six times a week for eight weeks. MI was performed concurrently with electrical stimulation and training was performed twice a week; each treatment lasted 40 min, and training was performed 12 times a week for six days over the course of eight weeks. Upper limb function and ability to perform daily tasks were assessed in three groups before and eight weeks after therapy. MIT combined with intramuscular adhesive was shown to significantly improve upper limb motor function and the daily living ability of stroke patients, and reduce abnormal upper limb tone when compared with conventional treatment.

#### 3.3.3 MI combined with traditional Chinese medicine

Zhang et al. (26) treated 18 stroke survivors with three-dimensional acupuncture at several acupoints, including the shoulder iliac, JiQuan, QuChi, WaiGuan, HeGu, HuanTiao, XiGuan, YangLingQuan, FengLong, SanYinJiao, and TaiChong. The blood supply point (1.5 inches below the Fengchi Point), NaoLing point, NaoZhong point, NaoShen point, and BiZhong point were used as the locations for the needle. Limb function was assessed both before and after a 30-day course of MI therapy with acupuncture. In another study, Xiang et al. (27) administered percutaneous acupoint electrical stimulation and MI treatment simultaneously to 41 patients with upper limb disability following stroke. Upper limb function was evaluated at two, four, and eight weeks into the treatment. Zhou et al. (28) provided routine rehabilitation training to 30 stroke patients for 40 min each time, emphasizing task-oriented training to enhance capacity for daily living. Each patient was instructed by the therapist to undergo MI training and therapy with an internal muscle-strengthening patch at the same time as the MI treatment. The individual patch approach was chosen, and each patch was left in place for 48 h before being changed once in two-dimensions (2D). Patients were trained twice a day, six days a week for eight weeks, and rehabilitation effects were assessed during and after therapy.

### 3.4 Application of MI therapy to rehabilitate upper limb motor function in stroke patients with hemiplegia

#### 3.4.1 Correcting learned disuse

Patients with hemiplegia who experience learned disuse stop using their limbs at both neurological and behavioral levels, thus preventing them from regaining limb function. Over recent years, Motor Imaging Therapy and Modified Compulsory Motor Therapy have been increasingly used in the rehabilitation of upper limb function after stroke. On top of the standard rehabilitation therapy, Sui et al. (29) added two supplementary therapies. The Modified Barthel Index (MBI), Fugl-Meyer Motor Function Scale, and Simple Upper Extremity Function Examination (STEF) scales among the four groups of patients tested were better than the control group after four weeks of treatment. The damaged upper limb had to be used because the healthy upper limb was restricted. In order to reverse “learned disuse,” the rehabilitation training should be completed at a certain intensity, and system and special functional activities should be performed according to the functional status of each patient. Subsequently, the functional restructuring of brain plasticity should be promoted and neuromuscular function should be improved.

#### 3.4.2 Reconstructed neural circuit

The primary goal of stroke rehabilitation is to address limb dysfunction, especially upper extremity motor function, as this can improve a patient’s quality of life. Ji et al. (30) applied visual motor imagination based on conventional therapy and myoelectric-triggered functional electrical stimulation. After six weeks of therapy, the FMA score, mean myoelectric value and MBI score of upper limb motor function in patients from the observation group were all higher than those in the control group. By practicing active conscious control, the coordination of active and antagonistic muscles could activate potential synapses of the central nervous system, forming new synaptic connections, generating new sensory excitation, rebuilding the nerve circuit, restoring upper limb motor function, and generally improving quality of life. Verification experiments were carried out by He et al. (31) and Qu et al. (32) and identical findings were generated. Additional confirmation was provided by Pu et al. (33) and Villa-Berges et al. (34). The combined effects of stereo acupuncture and motor imagination therapy were noticeably superior to those of either treatment alone, according to research conducted by Zhang et al. (26), who demonstrated that repeated regular stimulation helped to establish the corresponding reflex arc, thus improving motor ability in the affected limb in a rapid manner, and promoting nerve remodeling.

#### 3.4.3 Regulation of muscle tone

The effect of intervention in patients with hemiplegia after cerebral infarction is not ideal because traditional rehabilitation training can increase tension in the antagonist muscle while strengthening the patient’s muscle strength. According to Xiao et al. (35) the combination of motion imagination with isokinetic muscle strength rehabilitation training can increase the strength of the elbow extensor muscle while reducing tension in the elbow flexor muscle; this practice can facilitate coordinated movements of the upper limb and improve upper limb function. It is critical to rely on the coordination of shoulder, elbow, and hand functions to perform essential activities of daily living with the upper extremity. However, this particular study did not perform a thorough evaluation of shoulder, elbow, or hand function; only the elbow joint of the hemiplegic side was treated.

#### 3.4.4 Establishment of normal movement patterns

Previous research has shown that after a stroke, motor control is reduced and the degree of muscle co-contraction increases on the affected side. Researchers believe that graded MI may involve a variety of neural functional networks that exert a strong regulatory effect on the motor system, thereby promoting the recovery of motor function. Over recent years, various researchers have conducted in-depth research and analysis of the mechanism responsible for activating the cerebral cortex in specific stages of graded MI. Gu et al. (16) reported that coordination between the biceps and triceps in the upper limb on the hemiplegic side clearly improved after graded motor imagining training when compared to the control group. These results suggest that graded MI therapy can help the upper extremity move independently, facilitate the development of normal movement patterns, stop abnormal movement patterns, and improve motor control in upper extremity. According to Cao et al. (36), the rearrangement of damaged areas in the brain is essential for the recovery of motor function in hemiplegic stroke patients. Seeing and imagining activities can lead to the activation of mirror neurons, areas of the brain associated with action planning and execution, in an effort to compensate for motor deficits throughout functional reorganization.

#### 3.4.5 Controlling the movement of brain hemispheres

At present, there is a shortage of objective methodologies utilized in domestic investigations such as electrophysiology and functional imaging (fMRI, diffusion tensor imaging). Wang et al. (37) demonstrated that the neural patterns that were engaged during mental tasks were comparable to those produced during actual movements by investigating motor function and electrophysiology before and after intervention. One of the most successful strategies for stroke recovery is task-oriented training. To improve upper limb motor function after stroke recovery, motor imaging treatment can reduce excessive activity in both hemispheres and rearrange the motor network in the ipsilateral hemisphere. This concept was supported by Zhang et al. (21) in their motion imagination joint rehabilitation robot study.

## 4 Conclusion

In conclusion, stroke patients and their families endure significant financial, physical, and psychological burdens due to the impaired function of their upper limbs. Existing research indicates that early rehabilitation training plays a crucial role in improving limb function, reducing the incidence of disability, and enhancing a patient’s ability to perform self-care. While Mirror Therapy (MI) has been shown to effectively improve upper limb dysfunction in stroke patients and is considered to be very safe, there remain several controversial aspects that require further investigation. First, most studies involving MI therapy, both domestically and internationally, suffer from a lack of objective assessment criteria, sensory function evaluation, and small sample sizes. Second, while many studies have demonstrated the short-term efficacy of MI therapy for the improvement of upper limb motor function, there is limited research on its long-term effects in limb rehabilitation. Third, MI therapy lacks standardized treatment protocols, including a defined time frame. The delayed widespread adoption of MI therapy is partly due to the challenges involved in implementing certain aspects of this form of treatment. To advance the development of community and remote home rehabilitation programs for stroke patients with hemiplegia, particularly in hospitals and underserved areas, future research should standardize key aspects of MI therapy, including sample size, intervention duration, long-term outcomes, and optimized training protocols. This will ensure that more stroke patients can benefit from this promising treatment.
